# Use of Fine-scale Geospatial Units and Population Data to Evaluate Access to Emergency Care

**DOI:** 10.5811/westjem.2018.9.38957

**Published:** 2018-10-18

**Authors:** Katherine M. Joyce, Ryan C. Burke, Thomas J. Veldman, Michelle M. Beeson, Erin L. Simon

**Affiliations:** * Northeast Ohio Medical University, School of Medicine, Rootstown, Ohio; † Cleveland Clinic Akron General, Department of Emergency Medicine, Akron, Ohio; ‡ Kent State University, Department of Public Health, Kent, Ohio; § Kent State University, Department of Geography, Kent, Ohio

## Abstract

**Introduction:**

Time to facility is a crucial element in emergency medicine (EM). Fine-scale geospatial units such as census block groups (CBG) and publicly available population datasets offer a low-cost and accurate approach to modeling geographic access to and utilization of emergency departments (ED). These methods are relevant to the emergency physician in evaluating patient utilization patterns, emergency medical services protocols, and opportunities for improved patient outcomes and cost utilization. We describe the practical application of geographic information system (GIS) and fine-scale analysis for EM using Ohio ED access as a case study.

**Methods:**

Ohio ED locations (n=198), CBGs (n=9,238) and 2015 United States Census five-year American Community Survey (ACS) socioeconomic data were collected July—August 2016. We estimated drive time and distance between population-weighted CBGs and nearest ED using ArcGIS and 2010 CBG shapefiles. We examined drive times vs. ACS characteristics using multinomial regression and mapping.

**Results:**

We categorized CBGs by centroid-ED travel time in minutes: <10 (73.4%; n=6,774), 10–30 (25.1%; n=2,315), and >30 (1.5%; n=141). CBGs with increased median age, Hispanic and non-Hispanic Black population, and college graduation rates had significantly decreased travel time. CBGs with increased low-income populations (adjusted odds ratio [AOR] [1.03], 95% confidence interval [CI] [1.01–1.04]) and vacant housing (AOR [1.06], 95% CI [1.05–1.08]) had increased odds of >30 minute travel time.

**Conclusion:**

Use of fine-scale geographic analysis and population data can be used to evaluate geographic accessibility and utilization of EDs. Methods described offer guidance to approaching questions of geographic accessibility and have numerous ED and pre-hospital applications.

## INTRODUCTION

Geographic analysis is a highly relevant methodology for assessing spatial accessibility, i.e., access to and utilization of emergency departments (ED). This methodology requires careful selection of both geospatial units and data sources. Individual-level residential addresses and socioeconomic or health data provide the finest scale of analysis, although access to such data is often not possible. Using the state of Ohio, this study evaluated the benefits and limitations of using freely accessible, fine-scale geographic entities, socioeconomic data from the United States (U.S.) Census (five-year American Community Survey [ACS] estimates), multinomial regression and geographic information system (GIS) analysis to evaluate travel time from EDs in relation to demographic and socioeconomic population characteristics. Modification of these methods have numerous applications in emergency medicine (EM), including access of individual patients to any or a specific ED, market oversaturation, or establishing pre-hospital transport protocols.

### Geospatial Units

“Coarse” geospatial units include census tracts (CT), county, and ZIP codes. Use of coarse-scale geospatial units potentiates the risk for “ecological fallacy,” in which aggregate characteristics of a population within a given area incorrectly suggest characteristics of its subdivisions or individuals.^1^,^2^ “Fine” geospatial units include census block groups (CBG) and small area estimation. Many CBGs make up one CT. CBGs contain 600–3,000 people, while CTs contain 1,200–8,000 people. These units “do not cross state, county or CT boundaries but may cross the boundaries of any other geographic entity.”^21^ Fine-scale, freely accessible units such as CBGs have been used to analyze large population health datasets in a variety of contexts including childhood obesity, cancer patient outcomes, immunization patterns and numerous projects conducted by the Public Health Disparities Geocoding Project.^6^–^9^ Datasets that pair with such units are free and publicly available. ACS data at the CBG level, used in this analysis, is particularly useful for investigating questions relating to spatial accessibility.

### Practical Application of Fine-scale Geospatial Units

Reliance on coarse-scale rather than fine-scale geographic areas has shown negative implications on health, seen in the delayed discovery of elevated blood lead levels in children of Flint, Michigan, during 2015.^11^,^12^ Kaplowitz et al. showed that when compared to ZIP codes, use of CBG characteristics offered better specificity and sensitivity both in the identification of high-risk children as well as opportunities for better cost savings.^13^ Similar approaches could be undertaken in EM to identify high-risk groups and opportunities for saving not only costs but improving health as well.

**Figure 1:**
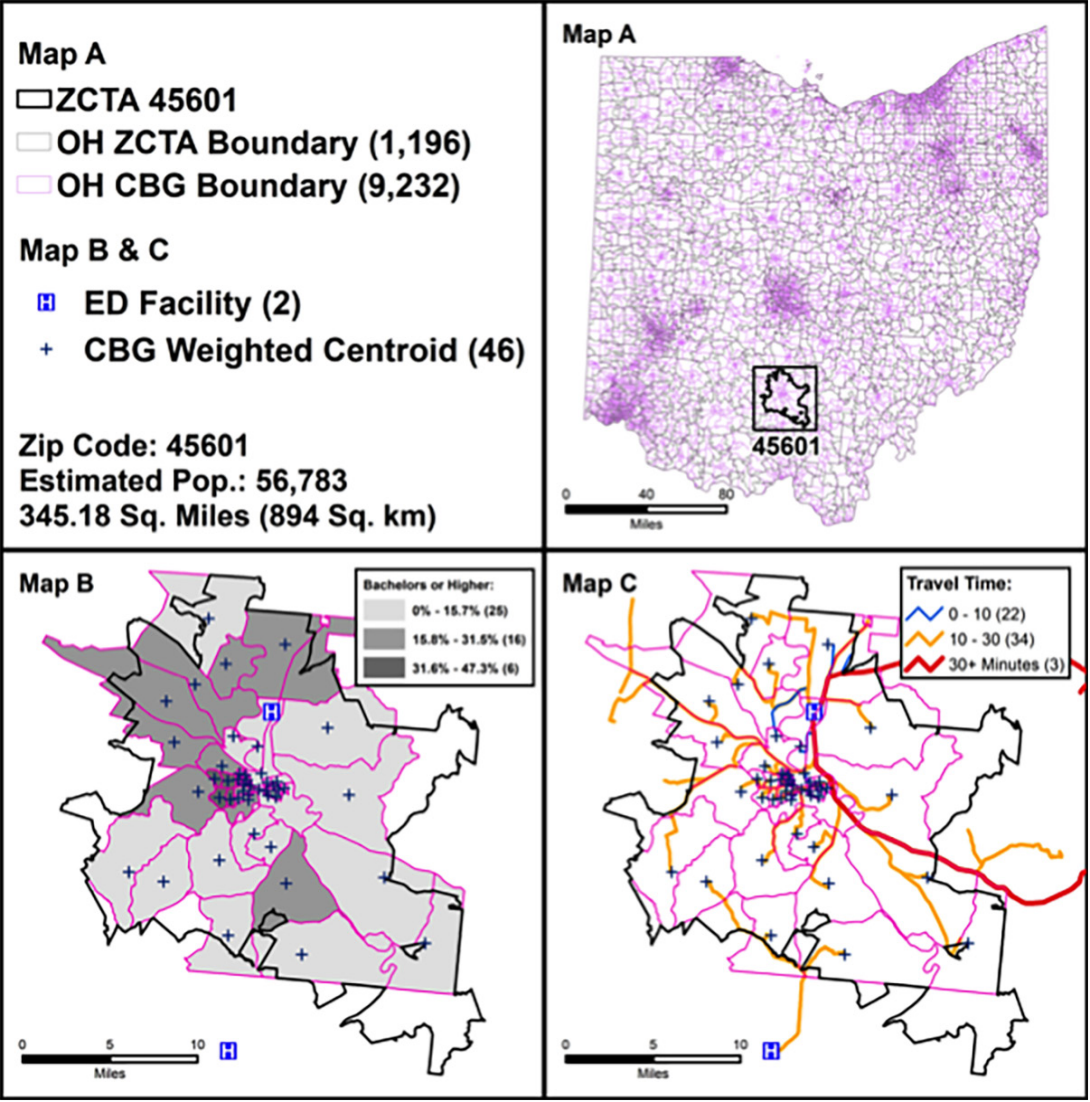
Maps A, B, C – Measuring emergency department access using census block groups vs. Zip Code tabulation area units.

Ohio census and ED data explain the difference between coarse and fine-scale units. Chillicothe, Ohio, and a branch of Ohio University sit within Ohio ZIP Code tabulation area (ZCTA) 45601 (3,458.18 square miles/894 square km). This is the largest Ohio ZCTA by area with a total population of 56,783 – approximately 40,000 of whom are age 25 and over (Figure 1, Map A). According to ZCTA units, 6,299 people (15.7%) of those 25 and over hold at least a bachelor’s degree. However, evaluating this characteristic using the CBG unit shows that this 15.7% is not uniformly distributed. Over half (25 CBGs) have only 0–15.7% of residents with a bachelor’s degree or higher. The remaining 22 CBGs (near a college campus) have a greater proportion of residents with a bachelor’s degree or higher. Similarly, estimates of distance of one CBG centroid to the nearest ED range from <10 min to >30 min between CBGs, giving a more nuanced picture of access to EDs (Figure 1, Map C). A similar phenomenon emerges in research regarding “hot spots” of violent crime, in which the majority of violent trauma incidents originate in a small geographic space.

**Population Health Research Capsule**

What do we already know about this issue?

Population health questions are often analyzed using “coarse scale” geographic units like zip code or census tract level data with Euclidean (as the crow flies) distances. Using coarse scale units can inaccurately represent a given population, particularly when studying access to emergency care.

What was the research question?

How does the use of fine scale geographic units and Manhattan distance impact analysis of access to emergency facilities?

What was the major finding of the study?

Use of fine scale geographic units and Manhattan distance in analysis of American Community Survey data yielded a more nuanced view of access to emergency departments in Ohio than would be possible with more coarse analytical units.

How does this improve population health?

Employing finer geographic units with a road network analysis, researchers can more accurately measure population characteristics and their overall level of access to healthcare services by modeling the actual paths one would use to access a facility.

### Access to and Utilization of Emergency Departments

Various studies have shown that increased time and distance to general or emergency clinical care increases mortality rates, making geographic accessibility to EDs important.^17^,^18^ Current guidelines recommend, for example, that if fibrinolytic therapy is to be used in reperfusion therapy of ST-elevation myocardial infarction patients, it be initiated within 30 minutes of hospital arrival.^16^

The state of Ohio has approximately 11.5 million residents and contains 1,197 ZCTAs, 2,952 CTs and 9,238 CBGs^22^, with 22.1% of Ohioans living in rural areas (89.2% of total area) compared to 77.9% living in urban areas (10.8% of total area).^23^ As of 2015, 198 EDs served Ohioans. This study evaluated distribution of and geographical access to those facilities by socioeconomic and demographic population characteristics. We used CBG population-level data from the 2010–2014 five-year ACS and geospatial analysis to help identify potential gaps in ED access. While the focus of this analysis was on demographic characteristics in a single state, similar methodology could be employed to analyze particular ED use patterns or pathologies.

## METHODS

We used free, publicly accessible resources to geocode addresses of individual EDs, incorporate a fine-scale geographic spatial unit (CBGs) within the state of Ohio, and use the most reliable socioeconomic data offered by the U.S. Census (five-year ACS estimates).

### Choosing Appropriate Geospatial Units in ED Access and Utilization Analysis

Apparicio et al. described four parameters required to properly measure geographic accessibility. Descriptions and the parameter chosen by this research group are described in Table 1.^5^ Road-network distances were particularly important, as most patients are transported to EDs by emergency medical services or personal vehicles. Time was considered more clinically appropriate than distance in determining access to services.

### Choosing Appropriate Population Data

This study used five-year data estimates extracted from the freely available U.S. Census Bureau’s ACS five-year estimate (2011–2015). ACS questions include general demographic questions, income, education and a variety of other socioeconomic factors. These data can be freely downloaded from American Fact Finder (https://factfinder.census.gov/faces/nav/jsf/pages/index.xhtml) or the National Historical Geographic Information System (https://www.nhgis.org). Other reasonable population datasets include electronic medical record or billing data. For purposes of a state-level analysis, ACS data seemed most appropriate to explain the methodology.

### Mapping

Individual street addresses of Ohio trauma centers and non-trauma center EDs (n=198) operating in 2015 were obtained and verified from publicly accessible state and federal databases and direct communications with administrators. We excluded psychiatric, veterans’ affairs and pediatric-exclusive EDs. Addresses were geocoded using Google Fusion Tables and Google Maps. CBG-level ACS population data and associated 2010 CBG shapefiles were acquired using the National Historic Geographic Information System (https://www.nhgis.org). We excluded CBG codes beginning with a zero (containing only water), located on an island and not connected to the main Ohio road network, or those with a null population (n=8). Maps used population-weighted centroids for CBGs (Figure 1, Map B) based on the population within each census block, as opposed to simple geographic centroids, which are less accurate in identifying where people live within a CBG. These population centroids acted as a proxy for patient address. Final analysis included n=9,230 population-weighted centroids, representing 2010 boundaries.

We modeled network drive times using Esri’s North American Detailed Streets network dataset (https://www.arcgis.com/home/item.html?id=f38b87cc295541fb88513d1ed7cec9fd). Once the network was established, centroid-ED distance and travel time were estimated using the closest facility function in Network Analyst and road-network (Manhattan) distance.^5^,^26^ We then stratified the centroid-ED pairs by travel time into three groups: <10 minutes, >10–30 minutes and >30 minutes. These categories were established based on literature linking increased mortality to these cut-points.^16^,^27^ We used CBG-level ACS data to assess statistically significant differences in relation to centroid-to-ED travel time. Multinomial regression was used to examine the association between travel time and CBG characteristics. We included variables significant at the univariate level in the multivariate model. All analyses were performed in IBM SPSS v21.

## RESULTS

**Figure 2:**
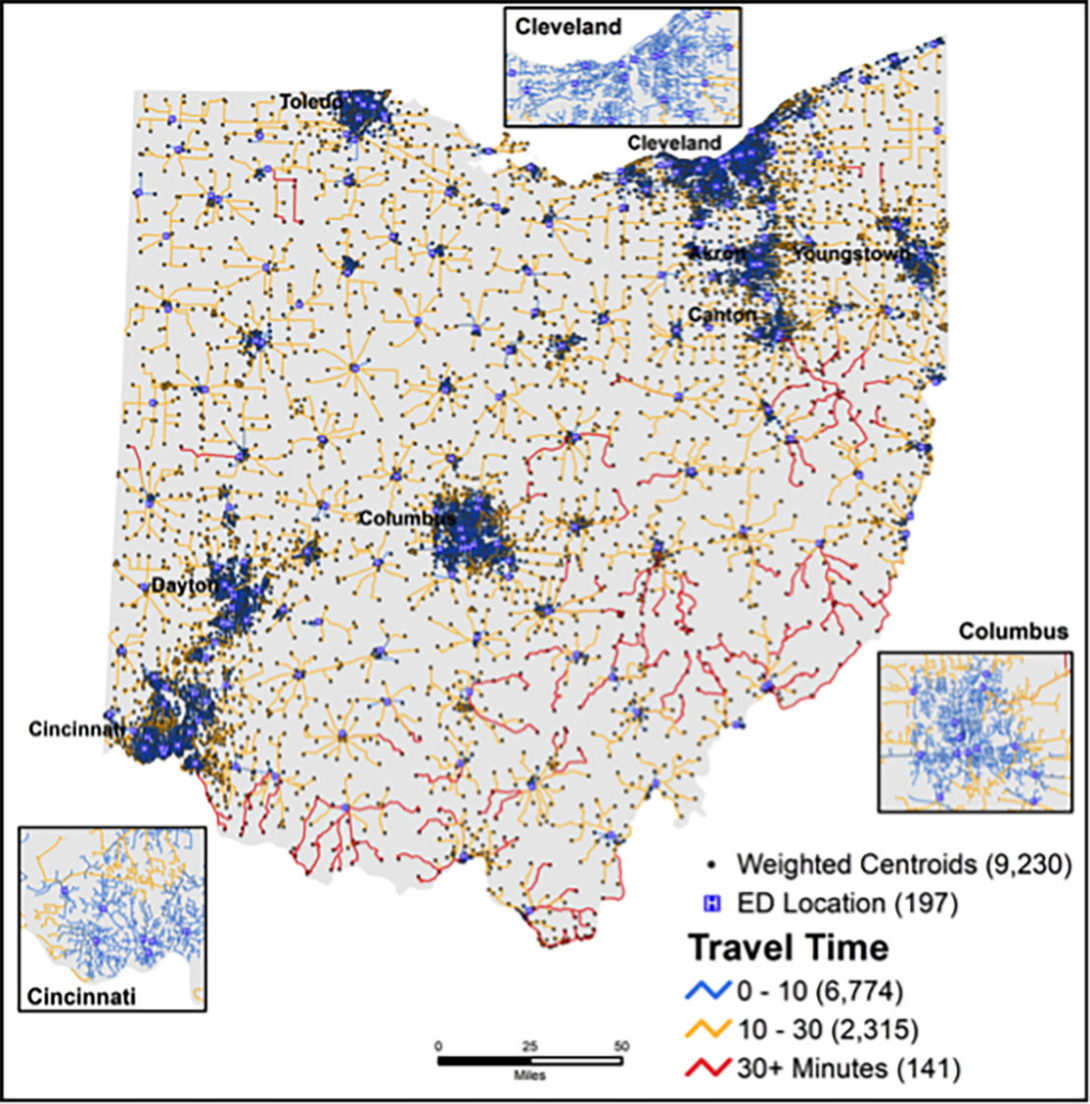
Population-weighted census block groups centroid to nearest emergency department (ED) travel time.

Of the 9,230 CBGs included in the analysis, 73.4% (n=6,774) had a <10 minute travel time. Of CBGs with increased travel time, 25.1% (n=2,315) had a 10–30 minute travel time, and 1.5% (n=141) had a >30 minute travel time (Figure 1, Map C). CBG population descriptions are presented in Table 2 and cartographic representation of travel times from CBG centroids to EDs is visualized in Figure 2.

Of the 15 CBG characteristics examined at the univariate level, only 10 remained significant in the multivariate model (Table 3). Travel time to the nearest ED significantly decreased with increased median age, increased Hispanic and non-Hispanic Black population, and increased college graduation rates.

## DISCUSSION

This study showed that the use of GIS, fine-scale geographic units, population data and network travel time to facility is an effective methodology to evaluate access to emergency care. The majority of Ohio CBG centroids had <10-minute travel time to an ED, and there appear to be minimal gaps in access among the population characteristics. Increase in a CBG’s median age, population density, percent Hispanic, non-Hispanic Black, educational attainment of at least a college degree, and owner-occupied houses had a decreased odds of having an increased drive time to an ED. As percent of a CBG’s population fitting these characteristics increased, odds of being farther away decreased.

While Ohio’s ED access appears to be generally robust, states with fewer medical facilities can use the methodology described to evaluate areas with a significant population requiring excessive transportation time to the detriment of the patient. It is also important to note characteristics of areas that have increased travel time. For instance, the odds of Ohio CBGs with increased low-income populations and vacant housing had an increased odds of being >30 minutes from the closest ED. Because a drive time over 30 minutes correlates with adverse patient outcomes, consideration of these CBGs when evaluating ED access is warranted.

This methodology may be used for densely populated areas to assess where “super-users” originate and establish targeted interventions to address these populations, thus reducing ED visits and costs while improving patient outcomes. Use of large datasets may also be useful in pairing patient-level data for clinical research or establishing disaster response protocols for emergency responders. For physicians and researchers with access to individual patient addresses in a given healthcare system or government, the described methodology can also be employed to create an even more nuanced picture of access and utilization of emergency care.

## LIMITATIONS

This study had several limitations. ED-specific characteristics, including available resources, proximity to higher level of care, patient volume, and average wait time, were not incorporated into analysis. While we calculated distance and time using accepted practice, the time travel models were made assuming no stops and at a fixed speed from origin to destination. This approximates but is not identical to real-world conditions, in which volume of traffic, stops at traffic lights, intersections, and weather conditions will add time between locations. Centroid-ED time was also based on residential address, rather than on where a patient was most likely to be at the time of an injury or illness. This is often a limitation regardless of dataset used, as patient address is often pulled from registration or billing data, which is tied to a patient’s home address.

## CONCLUSION

This study provides a guide for professionals interested in identifying the most appropriate population level data and geospatial units to identify gaps and opportunities in access to emergency care. Use of proper geographic and population characteristic tools is necessary to support individual patients and emergency medical staff as well as the systems they support.

## Figures and Tables

**Table 1:** The four parameters required to measure geographic accessibility parameters.

Description	Parameter selected
Spatial unit of reference for the population	Census Block Group
Aggregation method to account for distribution of population in residential area	Population-weighted centroids based on population within Census Blocks
Measure of accessibility	Travel time to closest emergency department
Type of distance for computing the accessibility measures selected	Road-network Cartesian (Manhattan)

**Table 2:** Ohio census block group characteristics, 2010–2014 United States. Census American Community Survey.

Characteristic	Mean (SD)	Median
Driving time to nearest ED (minutes)	8.3 (6.7)	6.2
Distance to nearest ED (miles)	4.8 (4.1)	3.4
Population density (per square mile)	3,119 (3,456)	2,167
Median age	40.2 (8.8)	40.2
Percent male	48.7 (6.2)	48.8
Race/Ethnicity (%)		
Hispanic	3.5 (6.7)	1
Non-Hispanic, White	78.4 (26.9)	90.3
Non-Hispanic, Black	14.2 (24.9)	2.1
Non-Hispanic, other	3.9 (5.3)	2.1
Education (%)		
No HS diploma/GED	12.5 (10.1)	10.1
HS diploma/GED/AA degree	64.4 (15.3)	67.6
At least a college degree	23.1 (18.7)	17.6
Income: Poverty Ratio <1.0 (%)	18.3 (16.9)	12.9
Unemployment rate (%)	6.4 (5.4)	5
Vacant houses (%)	11.4 (10.9)	8.9
Owner-occupied homes (%)	66.8 (24.5)	72.1
Household vehicle access (%)	90.6 (11.7)	94.9
Individuals without insurance (%)	11.6 (8.4)	10

*SD,* standard deviation; *ED*, emergency department, *GED*, General Education Development; *AA*, Associate of Arts.

**Table 3:** Results of a multinomial regression for travel time to the nearest emergency department.

Characteristic	10–30 vs. < 10 minutes		>30 vs. < 10 minutes	
	
	AOR	95% CI	AOR	95% CI
Median age	0.946	.937 – .954	0.967	.941 – .994
Population density	0.999	.999 – .999	0.998	.998 – .999
Percent Hispanic	0.974	.960 – .987	0.782	.693 – .884
Percent Non-Hispanic, Black	0.968	.961 – .975	0.911	.850 – .975
At least a college degree	0.975	.965 – .984	0.925	.897 – .955
Percent owner-occupied homes	1.02	1.015 – 1.025	1.027	1.011 – 1.044
Income: poverty ratio <1.0	0.993	.985 – 1.001	1.026	1.005 – 1.047
Unemployment rate (%)	0.987	.970 – 1.004	0.945	.899 – .994
Vacant houses (%)	1.007	1.000 – 1.015	1.064	1.047 – 1.080
Household vehicle access (%)	1.019	1.008 – 1.031	0.985	.958 – 1.012

*AOR,* adjusted odds ratio; *CI*, confidence interval.
